# A Novel COVID-19 Data Set and an Effective Deep Learning Approach for the De-Identification of Italian Medical Records

**DOI:** 10.1109/ACCESS.2021.3054479

**Published:** 2021-01-25

**Authors:** Rosario Catelli, Francesco Gargiulo, Valentina Casola, Giuseppe De Pietro, Hamido Fujita, Massimo Esposito

**Affiliations:** 1 Institute for High Performance Computing and Networking (ICAR), National Research Council 80131 Naples Italy; 2 Department of Electrical Engineering and Information TechnologiesUniversity of Naples Federico II 9307 80125 Naples Italy; 3 Faculty of Information TechnologyHo Chi Minh City University of Technology (HUTECH) Ho Chi Minh 723000 Vietnam; 4 Andalusian Research Institute in Data Science and Computational Intelligence (DaSCI), University of Granada 16741 18014 Granada Spain; 5 Faculty of Software and Information ScienceIwate Prefectural University 12835 Iwate 020-0611 Japan

**Keywords:** Clinical de-identification, contextualized embedding, deep learning, Italian language, named entity recognition

## Abstract

In the last years, the need to de-identify privacy-sensitive information within Electronic Health Records (EHRs) has become increasingly felt and extremely relevant to encourage the sharing and publication of their content in accordance with the restrictions imposed by both national and supranational privacy authorities. In the field of Natural Language Processing (NLP), several deep learning techniques for Named Entity Recognition (NER) have been applied to face this issue, significantly improving the effectiveness in identifying sensitive information in EHRs written in English. However, the lack of data sets in other languages has strongly limited their applicability and performance evaluation. To this aim, a new de-identification data set in Italian has been developed in this work, starting from the 115 COVID-19 EHRs provided by the Italian Society of Radiology (SIRM): 65 were used for training and development, the remaining 50 were used for testing. The data set was labelled following the guidelines of the i2b2 2014 de-identification track. As additional contribution, combined with the best performing Bi-LSTM + CRF sequence labeling architecture, a stacked word representation form, not yet experimented for the Italian clinical de-identification scenario, has been tested, based both on a contextualized linguistic model to manage word polysemy and its morpho-syntactic variations and on sub-word embeddings to better capture latent syntactic and semantic similarities. Finally, other cutting-edge approaches were compared with the proposed model, which achieved the best performance highlighting the goodness of the promoted approach.

## I. Introduction

In recent years, the availability of textual clinical data in electronic form, known as Electronic Health Records (EHRs), from which further information can be extracted to manage various critical health situations has become increasingly important. But, in order to be able to use such data, it is necessary to respect the restrictions imposed by both national and supranational privacy authorities: in the case of the USA the current law in force is the Health Insurance Portability and Accountability Act (HIPAA),^1^ in Europe there are the GDPR^2^ and several national legislations generally more restrictive but also less precise in indicating the exact procedures to follow. In principle, the removal of so-called Protected Health Information (PHI) through a process called de-identification is required before such health data can be made publicly available. Consequently, researchers are committed to improving de-identification methods also to help the world of medical research.^1^https://www.hhs.gov/hipaa^2^https://ec.europa.eu/info/law/law-topic/data-protection/data-protection-eu

According to HIPAA, there are two possible methods of de-identification: Expert Determination, which requires the employment of a human domain expert, and Safe Harbor,^3^ which can be automated as it defines 18 relevant identifiers that must be removed and/or replaced with plausible and realistic surrogates. The progress of such automated methods, which has gone from rudimentary rule-based techniques to techniques based on machine learning first and deep learning later, has been driven by the organization of several de-identification challenges by some international communities, such as i2b2^4^ and ShARe/CLEF eHealth Evaluation Lab.^5^ In this way the problem of de-identification has benefited from the use of Natural Language Processing (NLP) techniques such as Named Entity Recognition (NER), a task that aims to identify certain entities such as HIPAA identifiers. Unfortunately, these experiences remained confined to English, although there were some events related to other languages such as the IberLEF 2019 conference^6^ with the MEDDOCAN (Medical Document Anonymization) track in Spanish.^3^https://www.hhs.gov/hipaa/for-professionals/privacy/special-topics/de-identification/index.html^4^https://portal.dbmi.hms.harvard.edu/^5^https://clefehealth.imag.fr/^6^https://sites.google.com/view/iberlef-2019

This scientific article aims to make a scientific contribution regarding the positioning of the Italian language in the clinical de-identification scenario and, to reach this goal, three objectives have been pursued:

• the first objective consisted in the creation of a new data set for clinical de-identification in Italian proposed for the first time to the scientific community in this article: starting from the COVID-19 medical records made available to the public in pdf format by the Italian Society of Radiology (SIRM),^7^ the data were manually annotated according to i2b2 criteria [1];
• the second objective consisted in the construction, on the top of the best performing sequence labeling architecture recognized by scientific literature, i.e. a Bidirectional Long Short-Term Memory (Bi-LSTM) + Conditional Random Field (CRF) model [2], of a stacked form of word representation, not yet experimented for the clinical de-identification scenario in Italian, exploiting:
  • the Flair contextualized and character-level language model [3] to represent input words and respectively (1) capture the meanings associated to the same word in various contexts of use, i.e. the polysemy of the word, and (2) better grasp, interpret and manage both morpho-syntactic variations, i.e. the structures of words, such as endings and prefixes, and misspelled and/or rare words;
  • FastText sub-word embeddings [4] in order to better capture both the latent syntactic and semantic similarities;
• the third objective consisted in the execution of several experiments to verify the performance of the models previously described in comparison with BERT [5], a Transformer [6] based architecture, which is considered the state-of-the-art language model for many NLP general tasks and also the NER one [7], which includes the particular case of de-identification.

^7^https://www.sirm.org/

These tests have verified the effectiveness of different ways of functioning, for example statically or contextually and at character, sub-word or word level, on the Italian language which, even with an alphabet similar to the English one, presents a wide syntactic and morphological variety. In detail, the stacked embedding consisting of FastText and Flair has reached the best performance for the Italian de-identification scenario: the combined ability of handling context, polysemy and morpho-syntactic variations given by Flair and analysis at sub-word level given by FastText has surpassed the other models tested.

The remainder of this article is structured as follows. In Section II the most important works related to the topic and a general background are drawn. In Section III both the data set and the architecture used are described. In Section IV the experimental setup and the evaluation metrics are explained, while in Section V the results are analyzed and discussed. Finally, in Section VI, some conclusions and future works are discussed.

## II. Background and Related Works

In terms of information, a PHI can be assimilated to a *named entity*. The recognition of such entities occurs by implementing what is called NER, defined as *clinical* if applied on medical records in the form of unstructured text. The purpose is to be able to use the data contained in them, therefore it is necessary to identify the PHI and replace them with valid surrogates, a process called *anonymisation* [8]. For this reason it is important to recognize the type to which the entity belongs, and it would be more correct to refer to Named Entity Recognition and Classification (NERC).

Manual labeling of PHI, as stated by [9], does not allow either to reduce costs and errors related to human annotators or to outsource activity due to confidential data access. Therefore, automated systems have been developed that can be divided into two main categories, those based on rules and machine learning and those based on deep learning. More recently, these promising deep learning systems have been started being applied also to other languages different from English.

### A. Rules and Machine Learning Approaches

Starting from the first rule-based NER systems [10], several systems suitable for clinical use have been developed [11]–[15], all of them easy to implement and without the need of manual labeling. In fact, several automation tools have been created [12], used successfully [16] but hardly adaptable to languages other than English [17], [18].

Over the years, the effective but highly complex rules-based methods [19] have given way to machine learning systems where large amounts of data with easy-to-extract features were available for the training phase, [20]–[22] or to hybrid systems capable of detecting entities even in cases of scarcity of data and complex features, provided that they are more sophisticated and take more time to be implemented [23]–[26]. These machine learning (ML) algorithms have modeled the NER problem, i.e. de-identification, as a problem of classification [27], [28] or sequence labeling [29]–[31]. Among the latter emerged those based on Conditional Random Fields (CRFs), also in the de-identification field [22], accompanied by various feature engineering techniques [24], [25], [25], [26], [32], widely described in [33].

### B. Deep Learning Approaches (Embeddings and Language Models)

The usage of deep learning based systems [2], [34]–[36] improved the performances obtained for many NLP tasks and also for clinical NER [9], [37], exploiting two important elements: embeddings [38], that is a numerical representation of textual elements, and complex (deep) neural networks architecture [2], [34], [36], [39]–[41]. These findings have been applied to the clinical domain [42]–[45] then to de-identification [37], [46].

Embeddings are defined as vector representations of discrete variables such as words, characters or, even sentences. It is possible to obtain ready-to-use pre-trained embedding using large corpora. [4] have tried to change the way embedding works with interesting results: instead of associating embedding to words, FastText embeddings break them into sub-words, i.e. a set of characters that make up n grams, in order to reconstruct the embedding associated with a single word by looking at the various sub-word components identified. A similar approach has been used by BERT, whose tokenizer is based on WordPieceModel segmenter [47] which always works on a sub-word level. Over the years, these architectures have been improved to take into account, not only a static context (word2vec, glove, etc.) [38], [48] but also the relations among words within paragraphs (ElMo, BERT, GPT, etc.) [5], [49], [50] namely Statistical Language Models or, shortly, Language Models.

In details, BERT [5] and subsequent variants related to the biomedical world [51], [52], have paved the way for the use of techniques based on attention mechanisms [6]. Such techniques have been tested in different fields, such as chemical [53] or news [54].

Flair [3] have instead descended to the atomic level of text, seeing it not as a sequence of words or sub-words, but as a sequence of characters and adding to this contextual capability. This has resulted in state-of-the-art results in several NLP tasks.

### C. Clinical De-Identification for Specific Languages

Automatic de-identification and anonymisation systems in languages other than English, although lacking in language resources, have seen greater development in recent years. For example, in Danish, [55] have tried to balance the system in a way that both preserves readability and does not degrade the confidentiality of the large public EHR data set available. Also in Dutch, there have been developments: [56] were the first to test machine learning techniques, whereas [57] proceeded to compare even the most modern deep learning systems. In both cases it was necessary to request EHRs from Dutch institutes, which are often not publicly available. In French, both [58] and [59] explored the possibilities of rule-based and CRF-based systems on data sets built by retrieving EHRs from French hospitals. In German, first [60] and then [61] developed rule-based techniques and machine learning, but they remain proof-of-concepts due to the lack of extensive data training. In Norwegian, a rule-based method was developed by [62]. Also in Polish language there has been the development of some rules-based system [63], [64]. Rules-based systems in Portuguese [65] or machine learning in Swedish [66] were developed. Finally, in Spanish language there was the only other challenge organized besides the English language ones: the most recent MEDDOCAN: Medical Document Anonymization Track [67] within IberLEF 2019.^8^ As far as we know, there is no research on the subject in Italian to date.^8^https://sites.google.com/view/iberlef-2019

## III. Material and Methods

Below a detailed description of the data set, its pre-processing and annotation procedure is provided in Section III-A, while the Bi-LSTM+CRF architecture is described in Section III-B.

### A. SIRM COVID-19 Data Set

In this article the Italian SIRM COVID-19 data set, based on a collection of 115 unannotated medical records in pdf format released by SIRM,^9^ is developed. In order to proceed with the annotations, the guidelines adopted by [1] for the 2014 i2b2/UTHealth de-identification track were followed. In detail, the i2b2 2014 de-identification corpus was released by members of the i2b2 National Center for Biomedical Computing for the NLP Shared Tasks Challenges [33], and its annotation is consistent and enhanced against the Safe Harbor criteria. Indeed, the i2b2 project has further refined the 18 categories of PHI identifiers provided by HIPAA by expanding and then grouping them into 7 main categories and several subcategories.^9^https://www.sirm.org/category/senza-categoria/covid-19/

Finally, the SIRM COVID-19 data set was split: 65 medical records were used for training and 50 medical records for testing. To this end, the Table 1 presents an exhaustive list of PHI distributions in the SIRM COVID-19 de-identification corpus, with further details on training and testing entities. In the first column C:Subcategory, C: stands for the category to which the entities belong if present, in particular C, I, L, N stand for Contact, ID, Location and Name respectively. In detail, named entities are annotated by using subcategories as labels. Subcategories are then grouped into the appropriate categories as outlined by [1]. Some statistical data concerning the SIRM COVID-19 data set have been reported in Table 2.

**TABLE 1:** PHI Entity Distributions in the SIRM COVID-19 De-Identification Corpus. TR Stands for Training Data Set and TS Stands for Test Data Set

PHI C:Subcategory	TR	TS	Total
AGE	63	55	118
C:PHONE	3	7	10
C:URL	66	76	142
DATE	64	90	154
I:ID NUMBER	137	129	266
L:CITY	38	63	101
L:COUNTRY	1	5	6
L:HOSPITAL	134	132	266
L:ORGANIZATION	4	9	13
L:OTHER	3	6	9
N:DOCTOR	303	430	733
N:PATIENT	3	0	3
PROFESSION	38	27	65
PHI Category	TR	TS	Total
AGE	63	55	118
CONTACT	69	83	152
DATE	64	90	154
ID	137	129	266
LOCATION	180	215	395
NAME	306	430	736
PROFESSION	38	27	65
Total # of entities	857	1029	1886

**TABLE 2:** Statistical Data Concerning the SIRM COVID-19 Data Set

SIRM COVID-19 data set stats	
Average tokens per document:	262.8
Average NEs per document:	16.4
Average tokens per NE:	2.2

#### a: Pre-Processing

The data set has been annotated manually, generating the annotations in brat standoff format. In addition, several python scripts have been written to convert the data. First the pdf files were transformed into text using the python library *pandas*, then a python script was used to convert the brat standoff format to the CONLL format more suitable for the framework used, using as basis the publicly available NeuroNER tool [46] with spacy as tokenizer and *it_core_news_sm* as language model. To improve tokenizer results, entities have been separated from the rest of the text when wrongly attached, inserting a space before and after when appropriate. As a consequence, the misalignment caused between the initial and final offset values of the characters of the entity has been verified and adjusted within the text.

In the converted files, all entity labels are attached to the tokens according to the IOB tagging format [68] where $O$ represents all untagged tokens, $B$-tag represents the beginning of the label and finally $I$-tag is attributed to all the following tokens that still belong to the same named entity.

#### b: Annotation Procedure

The annotation procedure was carried out as described in the following. Each document was labeled manually and independently by three Italian native speakers, who are researchers in the e-health domain, with the agreement among the annotators calculated by majority. The global agreement for the entire annotation procedure was measured using the Observed Agreement index [69] which provides a good approximation in multi-annotator contexts, also offering robustness against imperfect (textual) data [70]. In addition to the Observed Agreement index, in order to take into account the level of Inter Annotator Agreement (IAA) in terms of excess over the agreement obtained by chance, the Krippendorff coefficient $\alpha $ [71] was also calculated. The latter expresses the IAA in terms of disagreement, observed ($D_{o}$) and due to chance ($D_{e}$): $\alpha = 1 - D_{o}/D_{e}$ and, not imposing a minimum number of items, mitigates the statistical effects of low sample data sets such as the one used. The Observed Agreement index value was 0.68, while the Krippendorff coefficient $\alpha $ value was 0.71: according to the grid for the interpretation of coefficients proposed by [72] the values obtained indicate a “substantial” agreement.

The disagreement among the annotators is generally motivated by the extreme difficulty, variety and uncertainty of natural language and, therefore, by a very diverse and often subjective linguistic understanding of the meaning of each category. In any case, disagreement is not strictly an indicator of low quality annotation, poor annotator training or insufficient guidelines, especially in semantic tasks [73], but can be used directly to improve the behavior of automatic systems [74], [75].

In Table 3 some sample sentences of disagreement among the three annotators have been reported. In particular in the *Sentence* column is reported the sentence under examination with the alternation of red and black colors to indicate different tokens, while in the macro column *Annotator sequence* are reported the annotation sequences for tokens of the first (*#1*), second (*#2*) and third (*#3*) annotator.

**TABLE 3:** Annotators’ Disagreement Examples

	Annotator sequence		
Sentence	#1	#2	#3
Uomo di 60 anni (Man of 60 years)	O O B-AGE O	O O B-AGE O	O O B-AGE I-AGE
ASL Latina Paziente Maschio (ASL Latina Male Patient)	B-HOSPITAL I-HOSPITAL O O	B-HOSPITAL I-HOSPITAL O O	B-HOSPITAL B-CITY O O
COVID-19: caso 98	O O O B-IDNUM	O O O B-IDNUM	O O O O
(COVID-19: case 98) (Arrives to the ER)
Giunge al PS	O O O	O O B-LOCATION_OTHER	O O B-HOSPITAL
Performance of radiologists (This is a test sentence)	O O O	O O B-DOCTOR	O O B-PROFESSION

### B. Bi-LSTMCRF System Architecture

One of the best performing sequence labeling architecture recognized by scientific literature is represented by the Bidirectional Long Short-Term Memory (Bi-LSTM) + Conditional Random Field (CRF) model, as demonstrated by [2], who tested several architectures such as LSTM, Bi-LSTM, CRF, LSTM+CRF and Bi-LSTM+CRF for sequence labeling task. In particular, Bi-LSTM+CRF is able to learn long-term dependencies exploiting both past and future input features thanks to a bidirectional LSTM component and, in addition, it can use sentence level tag information thanks to a CRF layer [2].

**FIGURE 1.**
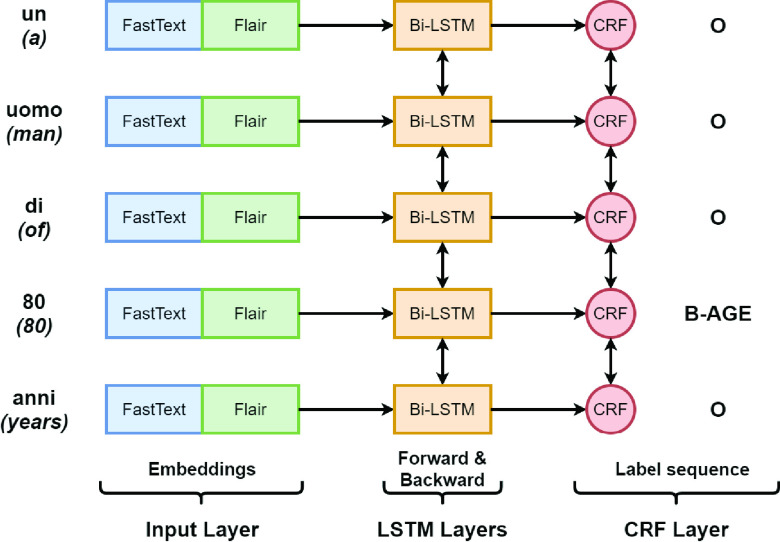
Bi-LSTM+CRF architecture overview.

The architecture overview of the proposed clinical de-identification system is shown in Figure 1 and detailed as follows.

Given to the LSTM an input sentence $(\textbf {x}_{1},\textbf {x}_{2},\ldots,\textbf {x}_{n})$ composed by n words, each represented as a $d$-dimensional vector, it is possible to obtain a representation $\overrightarrow {\textbf {h}_{t}}$ of the left context of the sentence at every word $t$. This is the output of the so-called forward LSTM. Using another LSTM that reads the same sequence in reverse, the so-called backward LSTM, it is possible to obtain $\overleftarrow {\textbf {h}_{t}}$. This couple of LSTMs is referred to as a bidirectional LSTM, whose superiority over unidirectional architectures for sequence tagging tasks such as NER has been widely demonstrated in literature [76] thanks to its ability to efficiently make use of both left (via forward LSTM) and right context (via backward LSTM) representations. The overall output is obtained by concatenating both left and right context representations: $\textbf {h}_{t}=[\overrightarrow {\textbf {h}_{t}};\overleftarrow {\textbf {h}_{t}}]$. Therefore, the representation of a word obtained using this model is an effective representation of a word in context. The equations implemented are the following:

\begin{align*} i(t)=&\sigma (\mathbf {W_{xi}}x(t)+\mathbf {W_{hi}}h(t-1)+\mathbf {W_{ci}}c(t-1)+b_{i}) \tag{1}\\[4pt] f(t)=&\sigma (\mathbf {W_{xf}}x(t)+\mathbf {W_{hf}}h(t-1)+\mathbf {W_{cf}}c(t-1)+b_{f}) \tag{2}\\[4pt] c(t)=&f(t)c(t\!-\!1)+i(t)\tanh (\mathbf {W_{xc}} x(t)+\mathbf {W_{hc}} h(t-1) + b_{c}) \\[4pt]\tag{3}\\ o(t)=&\sigma (\mathbf {W_{xo}}x(t)+\mathbf {W_{ho}}h(t-1)+\mathbf {W_{co}}c(t-1)+b_{o}) \tag{4}\\[4pt] h(t)=&o(t) \tanh (c(t))\tag{5}\end{align*}

where $\sigma $ is the logistic sigmoid function, and $c(\cdot)$, $i(\cdot)$, $f(\cdot)$ and $o(\cdot)$ are the cell vectors, the input gate, forget gate, output gate. The $\mathbf {W_{--}}$ matrices represents the matrices of weights calculated during the training. For instance, the notation $\mathbf {W_{xo}}$ represents the weight matrix of the input-output gate.

Then the modelling of joint tagging decisions happens through a CRF [29]. Given an input sentence **X**=(**x **_1_,**x **_2_,…,**x **_*n*_), an $n\text{x}k$ matrix **P** can be considered as the score output matrix of the Bi-LSTM. $n$ is the number of words contained by the input sentence, while $k$ is the number of distinct possible tags, hence $P {_{i,j}}$ is the score of the $j {^{\mathrm{ th}}}$ tag for the $i {^{\mathrm{ th}}}$ word of the sentence. For a sequence of predictions **y**=(**y **_1_,**y **_2_,…,**y **_*n*_), its score can be defined as

\begin{equation*} s(\textbf {X},\textbf {y})=\sum _{i=0}^{n} A {_{y {_{i}},y {_{i+1}}}}+\sum _{i=1}^{n} P {_{i,y {_{i}}}}\tag{6}\end{equation*}

where **A** is the matrix of transition scores, and A $_{i,j}$ represents the score of a transition from the tag $i$ to the tag $j$.

#### c: Embeddings

In this article we are focused on the following embeddings for Bi-LSTM+CRF system architecture:

• FastText Embeddings. Built and pre-trained over very large corpora by [4], these embeddings are statics related to context and work on the subword-level. In this way FastText embeddings attempt to capture morphological information to induce word embeddings and deal better with out of vocabulary words.
• Flair Embeddings. These embeddings are called *contextual string embeddings* by its creators [3]. These embeddings, pre-trained on large unlabeled corpora, combine several features: they are contextual hence produce different embeddings for polysemous words and they also model words and context by characters hence better managing misspelled and rare words and specific grammatical structure.

Moreover, as demonstrated by [51], the use of specific embeddings for the de-identification task does not provide improvements, so versions of embeddings trained on generic domains have been used.

## IV. Experimental Setup and Metrics

Hereafter the experimental setup and the evaluation metrics are described in Section IV-A and IV-B respectively.

### A. Experimental Setup

These experiments used Flair framework^10^ [77] for Bi-LSTM+CRF model implementation. It provides state-of-the-art general-purpose architectures with thousands of pre-trained models in over a hundred languages for NLP tasks, such as NER, part-of-speech (PoS) tagging, sense disambiguation and classification.^10^https://alanakbik.github.io/flair.html

Flair framework was used with the hyper-parameters reported in Table 4 and stochastic gradient descent (SGD) algorithm was used to estimate neural networks parameters. On the one hand we used only Italian FastText embeddings or only Flair (forward and backward) embeddings, on the other hand we stacked Italian FastText and Italian Flair (forward and backward) embeddings concatenating them.

**TABLE 4:** LSTM-Based Model Hyper-Parameters

Hyperparameter	Value
Annealing factor	0.5
Batch size	16
Dropout (Variational)	0.5
Dropout (Word)	0.05
Epochs	up to 500
Gradient clipping	5
Hidden size	256
Learning rate	from 0.1 up to 0.0001
Patience (early stopping parameter)	3
RNN Layers	1

**FIGURE 2.**
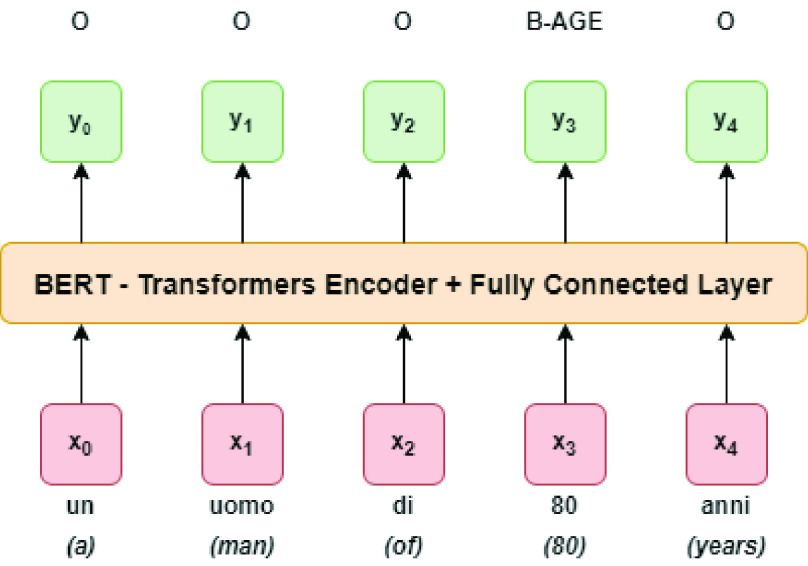
BERT architecture overview.

As far as we know, there are no other works for the particular NER task of clinical de-identification in Italian, since there are no publicly available Italian data sets. Hence, beside the Bi-LSTM+CRF model, the BERT model was tested too, which is another common state-of-the-art language model for different NLP tasks. In detail, the Hugging Face Transformers^11^ framework for BERT-based models was used, the main architecture is shown in Figure 2.^11^https://github.com/huggingface/transformers

**FIGURE 3.**
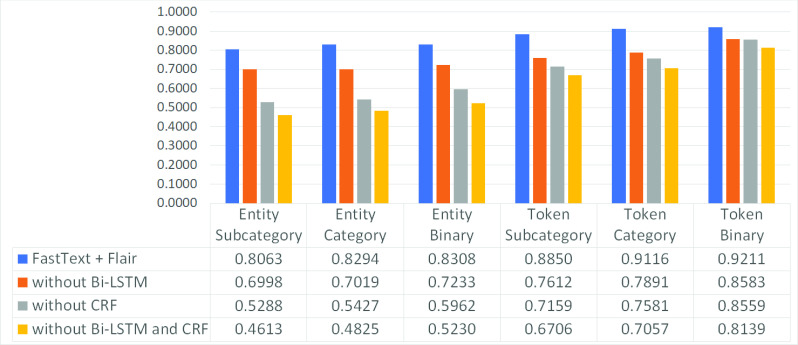
Ablation analysis.

In particular, the BERT architecture [5], which stands for Bidirectional Encoder Representations from Transformers, is a general purpose language model trained on a large text corpus (like Wikipedia), which can be used for various downstream NLP tasks, such as NER, Relation Extraction, and Question Answering, without heavy task-specific engineering. BERT $_{\mathrm {BASE}}$ architecture is based on 12 encoder layers, known as Transformers Blocks, 12 attention heads (or Self-Attention as introduced in [6]), and feed forward networks with a hidden size of 768. Instead, BERT $_{\mathrm {LARGE}}$ is based on 24 encoder layers, 16 attention heads and feed forward networks with a hidden size of 1024. For simplicity, if not specified, we will refer to BERT $_{\mathrm {BASE}}$ in the following.

BERT $_{\mathrm {BASE}}$ Maximum Sequence Length fixes the accepted embedding and encoder input/output vectors dimension to 512. Two special tokens are used: *[CLS]* and *[SEP]*. The *[CLS]*, which stands for *Classification*, is the first input token and produces an output vector of dimension equal to *hidden size* that can be used as the input for an arbitrarily chosen classifier. Instead, *[SEP]* stand for *segments separation*.

BERT, when used for NER, is fine-tuned without a CRF layer as output layer, following a diffused tagging task approach. Operating on the NER label set, the input provided to the token-level classifier uses the representation of the first sub-token. In detail, the final hidden representation $h_{i}$ of each token $i$ passes through the softmax function and the probability $P$ is then calculated as follows:

\begin{equation*} P(t|h_{i}) = softmax(W_{o} H_{i}+b_{o})\tag{7}\end{equation*}

where $t \in T$ while $W_{o}$ and $b_{o}$ are weight parameters. During training the loss function used is categorical cross-entropy.

In particular, the Italian BERT models used with the Hugging Face framework are those made available by the MDZ Digital Library team at the Bavarian State Library.^12^ For this study, the hyper-parameters shown in Table 5 were used. In detail, BERT-based models have $110 M$ of parameters. Batch size and Maximum Sequence Length were set to 32 and 512 respectively, while the model was fine-tuned for 5 epochs. Attention heads, hidden size and hidden layers were 12, 768 and 12 respectively. The Italian BERT was trained on a source of data consist made by a recent Wikipedia dump and various texts from the OPUS corpora^13^ collection with a final corpus size equal to about 13 GB and more than 2 billions tokens. Both the cased and the uncased versions were used.^12^https://huggingface.co/dbmdz/^13^http://opus.nlpl.eu/

**TABLE 5:** BERT-Based Model Hyper-Parameters

Hyperparameter	Value
Attention heads	12
Batch size	32
Epochs	5
Hidden size	768
Hidden layers	12
Maximum Sequence Length	512
Parameters	110 M

All experiments were performed on an IBM POWER9 cluster with NVIDIA V100 GPUs. All models were trained and tested using the chosen division of the data set between training and testing, reporting the results rounded to the fourth decimal place.

### B. Evaluation Metrics

In this article the performance metrics considered are micro-averaged Precision (P), Recall (R) and their harmonic mean, called F-measure ($F_{1}$).

From precision $P$ and recall $R$ it is possible to define their harmonic mean, called measure $F_{1}$ to evaluate the performance of the models and compare them. Said $TP$ the number of true positives, $FP$ the number of false positives and $FN$ the number of false negatives, we can define the metrics:

\begin{align*} F_{1}=&\frac {2*P*R}{P+R} \tag{8}\\ P=&\frac {TP}{TP+FP}=\frac {\#\;of\;correctly\;predicted\;items}{\#\;of\;predicted\;items} \tag{9}\\ R=&\frac {TP}{TP+FN}=\frac {\#\;of\;correctly\;predicted\;items}{\#\;expected\;items} \tag{10}\end{align*}

where items are entities or tokens, depending on the evaluation criterion used. In particular, according to the *entity* criterion, an entity is correctly predicted if it matches precisely the correspondent in the so-called gold standard, i.e. when all tokens belonging to it are correctly recognized. Instead, according to the *token* criterion, a token is correctly predicted if it matches precisely the correspondent in the gold standard, without considering the other tokens belonging to the entity it belongs to.

The results were produced using the criteria previously described, each of them divided into *binary*, *i2b2 category* and *i2b2 sub-category*. In the case of the *binary* criterion it is sufficient to discriminate between entities and non-entities (or tokens and non-token), then for the *i2b2 category* and the *i2b2 subcategory* it is necessary to recognize the categories and subcategories to which the entities or tokens respectively belong. So, *entity-subcategory* and *token-binary* level obtained the lowest and the highest scores respectively.

## V. Results and Discussion

The Micro-Averaged $F_{1}$^14^ scores of all tested models and related embeddings are shown in Table 6, ordered in accordance with the criteria given in Section IV-B.^14^Depending on how precision and recall are calculated, different types of $F_{1}$ can be obtained. In Micro-Averaging, the number of correct, predicted and expected entities or tokens of each class is added up and, with their total values, precision and recall are calculated. In Macro-Averaging, precision and recall values are calculated for each class, then overall precision and recall are calculated as the arithmetic average of class values. Instead, in Weighted Macro-Averaging, overall precision and recall are calculated as the weighted average (related to the number of expected entities or tokens for each class) of the precision and recall values.

**TABLE 6:** Micro-Averaged $F_{1}$ Results

Model	Embedding	Entity Level			Token Level		
		Subcategory	Category	Binary	Subcategory	Category	Binary
Bi-LSTM + CRF	FastText	0.7034	0.7130	0.7297	0.7821	0.8155	0.8395
Bi-LSTM + CRF	Flair	0.8100	0.8224	0.8289	0.8797	0.9045	0.9211
Bi-LSTM + CRF	FastText + Flair	0.8063	0.8294	0.8308	0.8850	0.9116	0.9211
BERT $_{\mathrm {BASE}}$ Uncased	–	0.6442	0.6667	0.6848	0.7667	0.8083	0.8796
BERT $_{\mathrm {BASE}}$ Cased	–	0.7553	0.7880	0.7969	0.8561	0.8979	0.9260

In regard to the Bi-LSTM + CRF model, FastText embedding, working at the sub-word level and managing semantic similarity accordingly, can better detect entities. Flair embedding, instead, relies more on its ability to exploit the context and manage polysemy. While individually FastText and Flair embeddings have comparable performance, a stacked embedding of their combination improves overall performance and is also the best method.

In addition, BERT $_{\mathrm {BASE}}$ Uncased achieves significantly lower results than the Cased version: this underlines the importance of training systems capable of distinguishing upper and lower case for clinical de-identification. In fact, in this sub-task of the NER, the Named Entities are often proper names, of people, places, or things, and therefore written with capital letters.

According to the results obtained, the Bi-LSTM + CRF model with the proposed stacked embedding (FastText plus Flair) performs better than all the others. It outperforms models made with FastText or Flair embeddings only and the BERT $_{\mathrm {BASE}}$ Uncased model. Instead, the BERT $_{\mathrm {BASE}}$ Cased model is outperformed in all metrics except one: it is important to underline that the BERT $_{\mathrm {BASE}}$ Cased model outperforms the Bi-LSTM + CRF model with the FastText plus Flair stacked embedding only at binary token level, the least significant to evaluate the performance of a NER system. It is of particular importance to consider this aspect in a de-identification scenario: in fact, the next step in this process is generally anonymisation, so it is necessary to obtain correct results at the most refined level of classification in order to replace the identified entities with valid surrogates [78], e.g. replacing a date with the surrogate of an ID number would allow the reader to easily identify the point of substitution by opening the door for an unwanted re-identification.

Therefore, although the data set is modest in size, using pre-trained embeddings and language models it is possible to obtain good performance. The Bi-LSTM + CRF model with the proposed stacked embedding made by FastText plus Flair showed superior performance compared to all other models analyzed: its detailed results are reported in Table 7. The subscripts ${E}$ and ${T}$ indicate Entity or Token level respectively.

**TABLE 7:** Detailed Results Obtained by the Best Model Bi-LSTM + CRF With Stacked FastText + Flair Embedding

i2b2 Sub-Category	$\mathrm {P_{E}}$	$\mathrm {R_{E}}$	$\mathrm {F_{1E}}$	$\mathrm {P_{T}}$	$\mathrm {R_{T}}$	$\mathrm {F_{1T}}$
AGE	1.0000	0.8909	0.9423	1.0000	0.8909	0.9423
CITY	0.4872	0.4191	0.4494	0.8452	0.4804	0.6108
COUNTRY	0.0000	0.0000	0.0000	0.0000	0.0000	0.0000
DATE	0.9022	0.7445	0.8151	0.9414	0.6914	0.7966
DOCTOR	0.8893	0.8312	0.8590	0.9825	0.8659	0.9203
HOSPITAL	0.6073	0.7955	0.6884	0.8891	0.9490	0.9180
IDNUM	0.9981	0.8140	0.8966	0.9981	0.6562	0.7919
LOCATION OTHER	0.5000	0.1000	0.1643	0.5000	0.1000	0.1643
ORGANIZATION	0.3186	0.3555	0.3305	0.8620	0.6000	0.6830
PHONE	0.9600	0.5428	0.6897	0.9600	0.5428	0.6897
PROFESSION	0.8773	0.5778	0.6960	1.0000	0.5692	0.7246
URL	0.9422	0.9421	0.9421	0.9974	0.8064	0.8918
Total	0.8323	0.7819	0.8063	0.9408	0.8355	0.8850
i2b2 Category	$\mathbf {P_{E}}$	$\mathbf {R_{E}}$	$\mathbf {F_{1E}}$	$\mathbf {P_{T}}$	$\mathbf {R_{T}}$	$\mathbf {F_{1T}}$
AGE	1.0000	0.8909	0.9423	1.0000	0.8909	0.9423
CONTACT	0.9426	0.9084	0.9251	0.9951	0.7881	0.8796
DATE	0.9022	0.7445	0.8151	0.9414	0.6914	0.7966
ID	0.9981	0.8140	0.8966	0.9981	0.6562	0.7919
LOCATION	0.6785	0.7080	0.6928	0.9499	0.9341	0.9419
NAME	0.8893	0.8312	0.8590	0.9825	0.8659	0.9203
PROFESSION	0.8773	0.5778	0.6960	1.0000	0.5692	0.7246
Total	0.8626	0.7986	0.8294	0.9691	0.8606	0.9116
i2b2 Binary	$\mathbf {P_{E}}$	$\mathbf {R_{E}}$	$\mathbf {F_{1E}}$	$\mathbf {P_{T}}$	$\mathbf {R_{T}}$	$\mathbf {F_{1T}}$
NAMED ENTITY	0.8664	0.7982	0.8308	0.9792	0.8697	0.9211

Analyzing the results obtained, it is possible to identify some aspects undoubtedly related to the type of data set. To better support this analysis, it is introduced in Table 8 the Token/Entity ratio (indicated as *T/E* in the Table for short) for each subcategory, calculated on the basis of the entities present in the data set and on how many tokens make up each entity.

**TABLE 8:** Token/Entity Ratio Per Subcategories

PHI C:Subcategory	# of Tokens	# of Entities	T/E
AGE	118	118	1.0000
C:PHONE	10	10	1.0000
C:URL	160	142	1.1268
DATE	200	154	1.2987
I:ID NUMBER	297	266	1.1165
L:CITY	139	101	1.3762
L:COUNTRY	6	6	1.0000
L:HOSPITAL	1297	266	4.8759
L:ORGANIZATION	56	13	4.3077
L:OTHER	9	9	1.0000
N:DOCTOR	1564	733	2.1337
N:PATIENT	3	3	1.0000
PROFESSION	96	65	1.4769

First of all, the *AGE* category is the only one to obtain high and identical results both at entity and token level: this is due to the general coincidence between the two levels, being the Token/Entity ratio equal to 1 in this case. Moreover all the entities are of numerical type, with few exceptions as for example the entity *sei* (six) and *47aa* (47yo).

The *CONTACT* category, although not as high and symmetrical, still obtains important results. In detail, this category is composed mainly of entities of type *URL* and minimally by entities of type *PHONE*. In particular, the entities of type *URL* can rely on rather repetitive patterns and, if broken on several tokens, on always the same introductory formulas (e.g. *http* and *www*). In the case of the entities of type *PHONE*, the only entity present is *118*: the subcategory is reduced in this case to a single numerical almost always recognized.

The *DATE* category, both at entity and token level, averages around a $F_{1}$ of 80%. Several considerations about the existing entities come into play here. The most often recurrent pattern is that of the type *gg/mm/yyyy* but not always in the same variant and for this reason it is not always identified: in some cases it is found *g/m/yyyy* or *gg/m/yyyy* or *gg.mm.yyyy* or *g/m* or *gg/m* or *yyyy - mm - dd*. Equally often there are the single entities *2020* or *marzo* and *febbraio* but it is often possible to find the English variants of the months of the year *January*, *February* or *Feb*, *March* and *April* or *April* because they refer to international studies of medical colleagues. Therefore the abundance of patterns not always numerous makes the recognition task less easy.

The category *ID* presents instead many mono-token entities introduced by the same formula (e.g. the numbers from 1 to 115 that indicate the medical records preceded by the pattern *COVID-19: caso* (COVID-19: case)) that contribute to keep the result especially high at entity level. However, the presence of a few scarcely recurrent if not unique and multi-token patterns lowers the performance at token level: in fact we have entities of the type *e200067*, *S2352302620301095*, *ehaa254* or multi-token as *10.1148/radiol.2020200823* in which the black-red alternation indicates the different component tokens.

The category *LOCATION* gets good results at token level but not entity. This behavior is generally due to the presence of several subcategories, such as *CITY*, *COUNTRY*, *HOSPITAL*, *ORGANIZATION* and *OTHER*. If for the entities of type *CITY* and *HOSPITAL* there is a sufficient number of samples more or less distributed between training and test sets, the same cannot be said for the other three categories, mainly present in the test set and with a small number of samples. In addition, for the *CITY* type entities there is an additional disadvantage due to the presence of a certain number of abbreviations, such as *VV*, *CE* and *VR* for *Vibo Valentia*, *Caserta* and *Verona* respectively, which are not very numerous and therefore difficult to recognize. On the other hand, for the HOSPITAL type entities there are tokens that are often repetitive components within the entities, as for example *UOC*, *ASST*, *AO* or *PO* even in the dotted versions, e.g. U.O.C., but the disturbing element is often the presence within the entity, as part of the hospital name, of entities that could also be indicated as *NAME* or LOCATION.

The category *NAME* achieves good results and in practice consists only of the subcategory *DOCTOR*. In this case, despite the token/entity ratio greater than 2, the results at entity level are not very far from those at token level. In fact, there are two recurring patterns: *Name Surname* or *N. Surname*, although in the latter case it may happen to find the entity constituted by a single token *N.Surname* which becomes more difficult to interpret, explaining the lower $F_{1E}$.

Finally, the *PROFESSION* category has the worst performance: this result is not unexpected as in NER tasks, and in de-identification tasks in particular [26], [37], it is quite common. This behavior is due to the peculiarity of this category: the professions are various and hardly recurrent in the medical records if used as a descriptive part of the personal information of patients, as for example *dipendente di un albergo* (hotel employee) and *medico di continuità assistenziale* (continuity of care doctor). On the other hand, to describe the roles in hospital facilities, if the medical records are rather sectorial as in this case, it is possible to find recurrent entities, such as *Direttore* (Director), which are always recognized.

### A. Qualitative Analysis

The Bi-LSTM + CRF model with the proposed stacked embedding made by FastText plus Flair works both at the sub-word level and at the character level exploiting the context: the results show that this proposed stacked embedding is particularly effective in improving the ability to detect and classify entities.

The presence of Flair embedding and its ability to work at character level allow the identification of a series of entities that FastText embedding alone is not able to detect, such as DOCTOR type entities where the surname is attached to the pointed name, such as *U.Burgio*, *M.Castiglia*, *L.Ferraro*, *M.Finazzo*, *G.Marsala*, *L.Putignano* and *A.Re*. Instead, the ability to exploit polysemy and context is effective both when entities are multi-token hence difficult to identify like HOSPITAL type entities such as *reparto di Osservazione Breve* (Short Observation Department), *U.O.C. di Malattie Infettive* (Infectious Disease Complex Operating Unit), *PO G. Di Cristina* (PO G. Di Cristina) and when entities are in foreign language, hence unusual, but mentioned in a specific context like DOCTOR type entities such as *Wang*, *Ruchong* and *Chunli*. In a similar way these capabilities make it easy to identify URL type entities such as *https://doi.org/10.3760/cma.j.cn112147-20200217-00106*, *https://doi.org/10.2214/ajr.20.22954* and *https://doi.org/10.1148/radiol.2020200823*. Some examples of polysemous entities are reported in Table 9.

**TABLE 9:** Examples of Polysemous Entities

i2b2 category: subcategory	Extracted sentence
LOCATION: HOSPITAL	Viene ricoverato inizialmente nel reparto di Osservazione Breve - Covid. (He was initially admitted to the Short Observation department - Covid.)
PROFESSION	presidio Ospedaliero di Vigevano, direttore ff reparto di radiologia Elena Belloni (Vigevano Hospital, director ff radiology department Elena Belloni)
LOCATION: HOSPITAL	UOC Radiologia Pediatria PO G. Di Cristina ARNAS Civico Palermo (UOC Radiology Pediatrics PO G. Di Cristina ARNAS Civico Palermo)
NAME: DOCTOR	Cristina Veirana, Alessandro Gastaldo UOC Radiologia, Ospedale San Paolo (Cristina Veirana, Alessandro Gastaldo UOC Radiology, San Paolo Hospital)

The use of sub-word level embedding, such as FastText, allows to identify semantically similar entities. In fact FastText embedding, unlike Flair one, is able to identify entities like *vibonese* and *lodigiano*: these are other ways to indicate the provinces of *Vibo Valentia* (often recurring as *Vibo*) and *Lodi* respectively and, although these entities are never seen before, their semantic similarities at sub-word level allow the system to recognize them. Similarly the entity *Veneto* when introduced by the term *regione* (region) is correctly recognized: in the training data set there is a similar introductory formula for another region, i.e. *Lombardia*. For the sake of completeness, the cosine similarity between entities are reported in Table 10.

**TABLE 10:** Cosine Similarity Between Words in Italian FastText Embeddings

Word 1	Word 2	Cosine similarity
vibonese	Vibo	0.50009230
lodigiano	Lodi	0.58718747
Veneto	Lombardia	0.62249140

The combination of Flair and FastText embeddings, despite the contextual capabilities, is not always able to recognize single token entities. Some examples in this sense are given by the entities *domenica*, *April* and *March* of type DATE, or by the entity *Reggio* of type CITY, as well as numerous DOCTOR type entities belonging to foreign but particularly short names such as *Han*, *Shi*, *Cao*, *Pan* and *Sun*.

It is interesting to note that there are some entities that are detected by the Bi-LSTM + CRF model with FastText plus Flair embeddings but not by BERT $_{\mathrm {BASE}}$ Cased model, and this is probably due to a different work at character and sub-word level: for example we have the *118* entity of type PHONE, or the *SOC Radiodiagnostica* (complex radiodiagnostic operating structure) entity of type HOSPITAL only partially detected by BERT $_{\mathrm {BASE}}$ Cased model.

Some significant examples of challenging entities for all the models have been reported in Table 11. In some cases, as for the entities of type PROFESSION *clinici* (clinics) and *dipendente di industria chimica* (chemical industry employee) the recognition is difficult due to the lack of examples in the training data set combined with ambiguities and complex patterns respectively. On the other hand, the HOSPITAL entity *reparto dedicato ai pazienti COVID-19* (ward dedicated to COVID-19 patients) is rather ambiguous and annotated in a questionable way, therefore difficult to identify. Finally, among CITY entities, it remains very difficult to recognize *VV* which is an abbreviation, albeit present in an extended form and with capitalized initials within the context.

**TABLE 11:** Examples of Unidentified Entities; in Blue are Identified the Entities Belonging to LOCATION Category Whereas in red the Ones Belonging to PROFESSION Category

$[\ldots]$ La Pz viene assistita e trattata in MU (reparto dedicato ai pazienti COVID-19)
e dopo circa 1 settimana dimostra notevoli miglioramenti $[\ldots]$
$[\ldots]$ The patient is assisted and treated in emergency medicine
(department dedicated to COVID-19) patients and after about 1 week
shows significant improvements) $[\ldots]$
$[\ldots]$ in accordo con i colleghi clinici del Pronto Soccorso con attribuzione di uno
score radiologico per quantificare l’estensione di malattia. $[\ldots]$
$[\ldots]$ in agreement with clinical colleagues in the ER with the attribution of a
radiological score to quantify the extent of the disease. $[\ldots]$
$[\ldots]$ esami ematochimici con PCR lievemente aumentata. Mamma
dipendente di industria chimica con casi positivi al tampone naso - faringeo. $[\ldots]$
$[\ldots]$ blood chemistry tests with slightly increased C-reactive protein. Mother
employee of chemical industry with positive cases of nose - pharyngeal swab. $[\ldots]$
$[\ldots]$ Giunge al PS di Serra San Bruno (VV) per riferita febbre (da almeno 5 giorni) $[\ldots]$
$[\ldots]$ He arrives at the emergency room of Serra San Bruno (VV) for reported fever
(for at least 5 days) $[\ldots]$

Entities of type *COUNTRY*, such as *Italy*, *Inghilterra* and *China* are not recognized by any system because of the lack of representativeness and disparities within data sets: in the training system we find only *Italia* of type *COUNTRY*.

### B. Ablation Analysis

The ablation analysis allows to understand the weight of the main components of a system within a given scenario [3], [9]. Here it can be seen which layer makes the greatest contribution to clinical de-identification in a low-resource language scenario with a small data set. In the specific it goes to compare a baseline, constituted by the best model that is the BiLSTM + CRF with FastText plus Flair embedding, with three ablated models: one will not have the CRF layer, the second one will have a simple Feed Forward layer instead of the BiLSTM layer and the third one without both CRF layer and Bi-LSTM layer (substituted by the Feed Forward one).

When the Bi-LSTM layer is replaced by a linear Feed Forward layer, i.e. when a multinomial logistic regression [79] is applied, then the label prediction is obtained as $P(\textbf {y}_{t} = j | \textbf {h}_{t}) = softmax(\textbf {h}_{t})[j]$ where the hidden layer $\textbf {h}_{t}$ is equal to $\textbf {W}_{h} \textbf {x}_{t} + \textbf {b}_{h}$.

This analysis allows to highlight two key aspects for this particular scenario:

• the combination of a BiLSTM layer and a CRF layer always achieves better performance than the individual layers;
• as the level of classification difficulty increases, it is possible to better distinguish the contributions of the different layers: in fact, if the CRF layer and the BiLSTM layer seem to have almost the same weight in a binary token scenario, the difference in favor of the model with the CRF layer becomes more evident proceeding towards the entity subcategory scenario.

To sum up, if on the one hand each removal has resulted in a marked reduction in performance suggesting that the choices made to assemble the analyzed architecture are correct, on the other hand it is possible to underline that, unlike what previously proposed by the scientific literature, it is not sufficient to conduct such a study limiting itself to the binary token layer as it could obtain misleading indications on the performance of the different layers composing the model.

## VI. Conclusion

In this study, a novel Italian data set was proposed for a challenging NER task, i.e. clinical de-identification. This data set was created from the COVID-19 medical records made available by the Italian Society of Radiology. It was labeled by three Italian native speakers and assessed by using two different indexes with a substantial agreement between them.

Moreover, a Bi-LSTM+CRF architecture in combination with a stacked embedding composed by FastText embedding plus Flair (forward and backward) embeddings was tested for clinical de-identification, on the proposed Italian data set.

Furthermore, another state-of-the-art architecture, i.e. BERT $_{\mathrm {BASE}}$, was tested leveraging the Italian models made available by the MDZ Digital Library team at the Bavarian State Library.

The Bi-LSTM+CRF architecture with the stacked embedding obtained the best results among the others. These results showed that it is desirable to adopt both contextualized and character-level language models in combination with sub-word embeddings: this way the system is capable to capture, on the one hand, the polysemy of words, their morpho-syntactic variations, rare words and/or misspelled ones and, on the other hand, the latent semantic and syntactic similarities.

In the future it might be interesting to compare other Italian versions of BERT or existing language models to see which ones are best suited for a clinical de-identification scenario and to assess if they can outperform the combination of the Bi-LSTM+CRF architecture with Italian FastText plus Flair stacked embedding herein tested.
